# Factors Related to Nutritional Status of Single Older Residents in Semi-Mountainous Rural Regions of Japan: A Cross-Sectional Study

**DOI:** 10.3390/geriatrics8020034

**Published:** 2023-03-05

**Authors:** Ai Nakai, Ikuharu Morioka

**Affiliations:** 1 School of Nursing, University of Kochi, Kochi 781-8515, Japan; 2 Graduate School of Health and Nursing Science, Wakayama Medical University, Wakayama 641-0011, Japan

**Keywords:** Mini Nutritional Assessment-Short Form, single older resident, rural region

## Abstract

Japan’s notably high aging rate presents the risk of malnutrition. This study aimed to clarify the nutritional status and factors related to the nutritional status of single older residents in a semi-mountainous rural region of Japan. Using a cross-sectional study design, surveys were administered to older adults in the semi-mountainous rural region in the area of Kochi Prefecture, Japan. Factors associated with a risk of malnutrition were identified using binomial logistic regression analysis. In addition, nutritional status was evaluated using the Mini Nutritional Assessment-Short Form (MNA-SF). Among 53 participants, the MNA-SF score was 12.1 ± 1.5 (mean ± standard deviation), and 71.7% had a normal nutritional status. We observed that participation in local residents’ association gatherings (odds ratio [OR]: 7.42, 95% confidence interval [CI]: 1.17–47.01) and risk of depression/anxiety (OR: 12.77, 95% CI: 1.99–81.94) were associated with an increased risk of malnutrition, whereas social interaction with friends (OR: 0.11, 95% CI: 0.02–0.76) were associated with a decreased risk. The nutritional status was normal overall. Community health workers should share information on the health of residents and promote social events to enable older residents living alone to continue leading healthy lifestyles.

## 1. Introduction

According to the United Nations World Population Prospects 2019, 28% of the population in Japan is aged 65 years or older (aging rate) [1], which is considered high. The population aging has shifted on a global scale [2], and the number of households with single older adults living alone independently, whether married, is increasing [3]. In such households, shopping and cooking become burdensome [4]; overall, interest in food has diminished. Moreover, single older adults living alone have decreased interpersonal interactions, decreased outing frequency, and decreased appetite owing to a lack of exercise [5,6]. Changes in body functions caused by aging, such as deterioration of vision, taste, and smell, may also be related to loss of appetite [7,8]. In addition, increased fragility and a reduced ability to absorb nutrients make older individuals prone to malnutrition [9,10,11].

Semi-mountainous rural regions, spanning from the edge of the plains to the mountainous regions, experience even greater rates of aging compared with those in urban areas in Japan [12], which is projected to continue increasing in the future. Furthermore, because these areas are underpopulated, few grocery stores are situated nearby. Consequently, residents experience “food access problems”, whereby purchasing food is inconvenient and difficult. These difficulties are especially apparent among the older community [12,13], and for this reason, support measures, such as the provision of community buses, home delivery services, and personal shopper services, are being provided. However, these solutions are currently inadequate because issues such as malnutrition are not properly addressed.

Optimal nutrition in the older population is important not only for the prevention and treatment of various illnesses [14], but also for promoting independence, which improves their quality of life and promotes healthy aging [15]. Therefore, various studies have been conducted to promote healthy aging thus far [16]; however, most of them were aimed at community residents. On the other hand, the available knowledge on single older residents in semi-mountainous rural regions of Japan is currently insufficient because few studies have been conducted on their nutritional status and associated factors [17]. Additionally, because the living environment, such as free time and the household, differs between older adults living alone and those not living alone, related factors may differ from those of community residents not living alone. Furthermore, the number of single older residents is projected to increase; therefore, gathering information on their nutritional status and related factors to promote health is vital.

Therefore, our research aimed to clarify the nutritional status of single older residents in a semi-mountainous rural region of Japan and the related factors, wherein we aimed to use the study findings to provide these residents with support in maintaining a healthy lifestyle in rural areas.

## 2. Materials and Methods

### 2.1. Design

This was a cross-sectional study using an anonymous self-administered questionnaire.

### 2.2. Setting and Participants

The area covered by the survey was the semi-mountainous rural region of the western area of Kochi Prefecture, Japan, which accounts for approximately 95% of Japan’s forests [18]. The researched prefecture has the second highest aging rate in Japan. The targeted area is one in which aging is relatively highly progressing in comparison to the overall prefecture.

The population has declined since peaking in 1960, with a population size of 1222 inhabitants in 2021. The aging rate is approximately 44% [18], and the proportion of single older households and older couples exceeds 40%. The geographical conditions dictate the location of dwellings; hence, isolation is likely and neighborly interactions are scarce.

In Japan, the Social Welfare Council is organized in all cities, towns, and villages. Together with local residents, volunteers, local welfare officers, and community health care professionals, members are engaged in various activities to allow local residents to live healthy and secure lives without being isolated from society. In the subjective area, the Social Welfare Council is active. Its members, including regional promotion staff, local resident association presidents, and community professionals, visit each region and host social events to create a space for older residents to gather. Additionally, they call on single older residents at risk of isolation to promote participation in the local residents’ association gatherings.

Some older people in the community may have had difficulty responding to the invitation to participate in this study because they cannot read owing to reading difficulties, etc. Thus, the researchers asked the representative of the Social Welfare Council to select local residents that the community health workers could interact with on a regular basis and to introduce the participants to this study. The participant selection criteria were as follows: individuals aged 65 years or older, living alone, and with adequate verbal literacy. The survey was conducted using a self-administered questionnaire; however, because it was assumed that some participants would experience reading and writing difficulties, participants were invited to hosted events to complete their responses.

Although participants had already provided consent to participate based on the explanation from the Social Welfare Council representatives, on the day of the survey, willingness to participate was confirmed after another verbal and written explanation of the research purpose and methods by the researchers. After consent was confirmed, the questionnaires were distributed, and participants were asked to complete them; the researchers only assisted those experiencing difficulty in answering.

### 2.3. Ethical Consideration

The researcher verbally explained the aims and procedures of the study to participants and also provided these explanations in writing. This stated that personal information and answers would be protected, participation was voluntary, and there would be no disadvantage if the individuals decided not to participate. After obtaining their consent, the researcher handed out an anonymous questionnaire and collected it after recording the participants’ responses. Submission of the questionnaire was considered consent to participate in the study. This study was approved by the University of Kochi Ethical Committee (No. 20-38).

### 2.4. Measures

#### 2.4.1. Attributes

Based on an earlier study [4,5,6], sex, age, occupation, period of residence, marital status, parental status, presence or absence of children living within the prefecture, and medical history were noted. There were four occupation categories: retired, self-employed, part-time, or others. There were five categories for period of residence: less than 1 year, between 1 and 2 years, between 3 and 9 years, between 10 and 19 years, and more than 20 years. Marital status had two categories: never married or married, including divorce and bereavement. There were two categories for presence or absence of children living within the prefecture: within the same prefecture and out of prefecture. The medical history was noted, and those who had affirmative responses were asked to indicate whether they had hypertension, diabetes mellitus, heart disease, ophthalmic disease, pulmonary disease, musculoskeletal diseases, or others, with multiple answers possible.

#### 2.4.2. Nutritional Status

In this study, the Mini Nutritional Assessment-Short Form (MNA-SF) was used to evaluate nutritional status. This scale, created by Guiguz in 1996 [19], is widely used worldwide as a useful tool for assessing the nutritional status of older residents in the community [20,21]. The scale is scored by adding the scores of six items: body mass index and weight loss (anthropometric measurements), mobility (global assessment), food intake, neuropsychological problems, and acute disease (dietary questionnaire and subjective assessment). Body mass index was calculated using self-reported height and weight. In MNA-SF the range of scores was 0–14: scores of 12–14 points indicate normal nutritional status; 8–11, risk of malnutrition; and 0–7, malnourished status.

#### 2.4.3. Daily Living Activity

Since the participants of our study were older individuals living alone, the Instrumental Activities of Daily Living (IADL) was used to evaluate complex activities among daily activities. The IADL was developed by Lawton and Brody in 1969 [22] and consists of eight items: ability to use the telephone, ability to go shopping, food preparation, housekeeping, laundry, mode of transportation, responsibility for own medication, and ability to handle finances. Scores for males are between 0 and 5 points and those for females between 0 and 8, wherein higher scores indicate higher independence.

#### 2.4.4. Social Situation

Participants were asked about their community activities, participation, social interaction, and meal sharing. They were asked if they attended community activities such as seniors’ club activities, hosted events, local residents’ association gatherings, exercise classes, nutrition classes, or others, with multiple answers permitted. Furthermore, participants were asked about their social interaction, such as with neighbors, relatives, friends, and community health workers provided, for which multiple answers were permitted. Finally, they were asked to answer “yes” or “no” to the question, “Do you have someone you could share a meal with?”

#### 2.4.5. Environment

Participants were asked about how they received nutritional information, how frequently groceries were purchased, where groceries were purchased, the means of transport used to go shopping, and their subjective access to grocery shopping. Participants could select television, radio, newspapers/brochures, neighbors, word of mouth, and others, with multiple answers permitted for sources of information on food and nutrition. Grocery buying frequency could be selected as weekly, two to three times weekly, or more than four times per week. In addition, grocery shopping locations were chosen from traveling sales vendors, supermarkets, convenience stores, delivery services, grocery stores (smaller and more limited in the range of merchandise than a supermarket), or others. The means of transport used to go shopping included on foot, by bicycle, by car, by taxi, by community bus, none (do not go owing to delivery service usage), and others. In these two question items, multiple answers were possible. Subjective access to grocery shopping was defined as grocery shopping difficulty and investigated using a four-point Likert scale of difficult, somewhat difficult, somewhat easy, and easy.

#### 2.4.6. Psychological State

Participants were asked about their subjective health, subjective happiness, financial situation, and the Japanese version of the Kessler Psychological Distress Scale (K6) was used. Subjective health was investigated on a four-point scale of completely unhealthy, somewhat unhealthy, somewhat healthy, and healthy. Similarly, subjective happiness was examined on a four-point scale of completely unhappy, somewhat unhappy, somewhat happy, and happy. The financial situation was investigated on a five-point scale: very dire, somewhat dire, normal, somewhat comfortable, and very comfortable. The K6 was developed by an American, Kessler, in 2002 [23] and has been officially translated into Japanese, ensuring sufficient reliability and validity [24,25]. The K6 is a psychological scale developed for screening for mental health issues among adults in the general population. It is a six-item measure of psychological stress (depression/anxiety) that has occurred in the past 30 days. Questions are answered on a five-point scale from “always” to “never”, with a score of 0 to 4 for each item (0 to 24 total points). Based on research indicating that more than 5 points is equivalent to a psychological stress reaction [26,27], the current study designated scores of 5 points or more as indicative of a risk of depression/anxiety.

### 2.5. Statistical Analysis

The analysis was conducted without separated sexes to examine the nutritional status of older adults living alone in a semi-mountainous rural region since there was no significant difference in MNA-SF scores between males and females. First, the distribution of the MNA-SF scores was checked, and the Shapiro–Wilk test was conducted as a test of normality. In addition, to examine the factors related to malnutrition according to the MNA-SF, participants with a normal nutritional status were categorized as the “Normal nutrition group” and those at risk of malnutrition and with a malnourished status as the “Risk of malnutrition group”. Furthermore, to investigate the related factors, nonbinary items from participant characteristics, social situation, environment, and psychological status were converted to binary values (Table 1). The IADL was divided into the normal (independent) and low groups with median values by sex as the cutoff values. A chi-square test was used to determine the association between the binary factors and the MNA-SF categories. Binomial logistic regression analysis (forward selection based on likelihood ratio tests) was performed, with the significantly related items in the chi-square test as independent variables and the MNA-SF categories as dependent variables. Spearman’s correlation coefficient was calculated to confirm multicollinearity before using the independent variables. Statistical analysis was performed using SPSS version 28, in which the significance level was set at 5%.

## 3. Results

### 3.1. Participant Characteristics

Fifty-three participants were recruited by the Social Welfare Council representatives, all of whom agreed to participate (there were 53 valid respondents in this study). Participant characteristics are shown in Table 2. There were 7 male and 46 female participants, wherein forty-six (86.8%) participants were aged 75 years or older with an average age of 83.9 (standard deviation, SD ± 6.8) years. Of all the participants, 84.9% were retired, and 9.4% were self-employed. Additionally, 90.6% had been residents for 20 years or more. Among the 53 participants, 48 (90.6%) were married, and 46 (86.8%) had children; of those 46 with children, 36 (78.3%) had their children living in the same prefecture. In terms of medical history, 84.9% of the participants had a medical condition: 73.3% had hypertension, 13.3% had diabetes mellitus, and 11.1% had heart disease.

The average MNA-SF score was 12.1 (± 1.5). The normal nutrition group had 38 (71.7%) participants, and the risk of malnutrition group had 15 (28.3%). The median IADL was 4 (interquartile range [IQR] 3–5) for males (*n* = 7) and 7 (IQR 7–8) for females (*n* = 45), indicating that most participants were independent. Scores for depression/anxiety (K6) were significant in the Shapiro–Wilk test (*n* = 50) with a median of 3 (IQR 1–7.3), and 22 (44.0%) participants had a score of 5 or above, suggesting a risk of depression/anxiety.

The results for social situation indicated that 25 (47.2%) residents participated in the seniors’ club, 24 (45.3%) in the hosted events, 14 (26.4%) in the local residents’ association gathering, 7 (13.2%) in exercise classes, and 2 (3.8%) in nutrition classes. Contrarily, ten (18.9%) participants did not participate in any of the community activities. Furthermore, 29 (54.1%) participants enjoyed neighborly relations; 26 (49.1%), relationships with relatives; 21 (39.6%), friendships; and 12 (22.6%) were familiar with the community health workers. However, 15 (28.3%) participants indicated having no social interaction.

Seventeen (32.7%) participants said that they had someone to share a meal with, and 35(67.3%) said that they ate alone. In terms of environment, television was the most common source of information on nutrition (*n* = 37, 69.8%), followed by neighbors (*n* = 17, 32.1%) and newspapers and brochures (*n* = 16, 30.2%). Twenty-nine (54.7%) participants reported buying for groceries one time or less per week, of which supermarkets were the most common location for shopping (*n* = 39, 73.6%), followed by grocery stores (*n* = 16, 30.2%) and delivery services (*n* = 8, 15.1%). The most common means of transport used to go shopping was by car (*n* = 16, 30.2%), followed by transport by community bus (*n* = 10, 18.9%). Transport on foot, by bicycle, and none garnered five (9.4%) responses respectively, and transport by taxi three (5.7%). Thirty-five (68.6%) participants said their subjective access to grocery shopping was easy. Thirty-eight (71.7%) respondents rated their subjective health as healthy; 35 (71.4%), their subjective happiness as happy; and 47 (90.4%), their financial situation as comfortable.

### 3.2. MNA-SF-Related Factors According to Chi-Square Test

Results from the investigation on the relationship between participant characteristics, social situation, environment, psychological state, and MNA-SF categories (Table 3) indicated significant associations between participation in local residents’ association gatherings, social interaction with friends, social situation, and risk of depression/anxiety, psychological status, and MNA-SF categories. In the risk of malnutrition group, 71.4% of participants had the risk of depression/anxiety, with scores 38 points higher than the those of the normal nutrition group.

### 3.3. MNA-SF-Related Factors According to Logistical Analysis

Binomial logistic regression analysis was performed using the significantly associated items, local residents’ association gatherings, social interaction with friends, and risk of depression/anxiety, as independent variables. Prior to the input of independent variables, the absence of strong correlations in excess of 0.7 between independent variables in the correlation matrix was confirmed.

The results of the logistic regression analysis are shown in Table 4. Significant associations were shown for local residents’ association gatherings, social interaction with friends, and risk of depression/anxiety. Participation in the local residents’ association gatherings and the risk of depression/anxiety promoted malnutrition, whereas social interaction with friends mitigated it.

The results of the chi-square test on the model indicated that each variable was significant at <0.001. Additionally, the Hosmer–Lemeshow test results indicated a model fit (*p* = 0.850) with a deterministic rate of 82.0%; there were no outliers where the expected value exceeded the actual measurement by ±3.

## 4. Discussion

### 4.1. Characteristics of the Participants

More than 90% of the single older residents living in the semi-mountainous rural region were in the late stages of aging, and most had lived there for more than 20 years. Thus, participants were considered to be accustomed to life in the natural physical environment of the semi-mountainous rural region. Because most of the participants had been married and had children living in the same prefecture, they appeared to be able to engage with their children daily, despite living alone at present. Moreover, the IADL indicated independence, with many individuals managing their health conditions. These results are similar to those indicating that many married older people living within the community also manage their health conditions [28,29].

Approximately 20% of respondents did not participate in community activities, and 30% did not have social interactions. Consequently, many people were in an environment that promoted isolation. Without community activities or social interactions, opportunities to share a meal decrease [30]; 30% of the respondents did not have the opportunity to share a meal. Many people used cars as transport to the supermarket to do their weekly shopping, and many expressed that access to food was easy. However, a limited number of participants used the community bus or taxi as transport for shopping purposes; thus, food access may be strongly related to the physiological and psychological factors of single older people rather than just the geographical and environmental factors [31].

### 4.2. Nutrition Characteristics of the Participants

According to the MNA-SF nutrition assessment, 71.7% of people had normal nutrition. An earlier survey of older people in Poland (*n* = 273) revealed an MNA-SF score of 11.8 ± 2.5 [32]. Moreover, 63% of the older people in community nursing homes had scores of less than 12 points, and 40% were at risk of malnutrition, with scores below 7 points [33]. In both studies, less than half of the respondents had normal nutrition. By contrast, in the current study, many of the late-stage older people had normal nutrition despite predictions of malnutrition. This suggests that living in the semi-mountainous rural region involves independence with regard to daily living activities, preparation of one’s own meals, and having normal nutrition; without these factors, older residents may have relocated from the semi-mountainous rural region, becoming reliant on their children. Therefore, normal nutrition may be a result of continued independent living.

### 4.3. Relationship between MNA-SF and Social Situation and Psychological State

Social situation items, such as participation in local residents’ association gatherings and social interaction with friends, were related to the nutritional status of single older people, as was the psychological state item of risk of depression/anxiety. Reduced neighborly interaction is associated with low fruit and vegetable intake among older people living alone [34]. Social connections are likely to positively affect nutritional status because they reduce isolation and increase the opportunities for older people to go outdoors and exercise [35,36].

Local residents’ association gatherings are open to all residents, and anyone is welcome to attend them. We initially assumed that older people who attended these gatherings were in good health, i.e., well-nourished, which would allow them to be able to participate. In this study, however, participation in the gatherings was a factor associated with malnutrition. In the area covered by this study, information on residents is shared between community health workers, regional promotion staff, and the local residents’ association presidents, and older people who are isolated and at risk of malnutrition are actively targeted and encouraged to participate in community activities. As such, individuals with a high risk of malnutrition are likely to be involved in the gatherings. Community health workers’ proactive involvement in the residents’ health conditions was considered a characteristic aspect of the semi-mountainous rural region. This study did not investigate whether meal-sharing opportunities were a part of the local residents’ association activities. Considering that 32.7% of the participants said they had opportunities for meal sharing despite living alone, we suggested that such activities should be investigated in the future.

For older individuals, social interaction with friends is actively pursued on their own; however, certain physical and mental health conditions are required to maintain high levels of social activity [37]. Thus, normal nutrition is considered necessary to facilitate social behavior. The likelihood of depression is 31% higher (odds ratio = 1.311) in people with malnutrition than in those with adequate nutrition [38]. This is consistent with our results that the likelihood of risk of depression/anxiety was 38 points higher in the risk of malnutrition group than in the normal nutrition group. Our results indicated an association between malnutrition and risk of depression/anxiety. This also corresponds to the results of an earlier study in which depression was identified as a related factor for malnutrition/risk of malnutrition in older adults requiring long-term care [39].

### 4.4. Further Mutual Support

The proactive involvement of community health workers, regional promotion staff, and resident association officials appears to be successful in preventing malnutrition for at-risk community members. Although the association was not apparent in the current research owing to the high number of independent participants with normal nutrition, there is a significant association between low ADL and low MNA-SF in older residents [30]. Moreover, because mental disorders such as depression in single older residents are related to low nutrition [40], community health workers should promote hosted events and other activities to raise awareness of physical and mental health issues. This may aid older individuals in the semi-mountainous rural region in maintaining a healthy lifestyle into old age. In the future, further knowledge should be gathered to realize a sustainable community in the semi-mountainous rural regions of Japan, which have high aging rates and increasing rates of depopulation.

### 4.5. Limitations

Our study had several limitations. Firstly, the sample size was insufficient to analyze the data because there were few residents, even in areas with a high aging rate. Despite the low number of cases, the results continue to serve as a starting basis to potentially be built on as more data are collected, and we foresee these results becoming a fundamental template for the construction of a comprehensive community care system in semi-mountainous areas where the number of older people living alone is expected to increase in the future. Secondly, participants were recruited via the Social Welfare Council and from among older residents who participated in hosted events. Therefore, the health of the participants was predicted to be good, even among older residents living alone, because they could participate in community events, which may have resulted in some bias regarding participant characteristics. Thirdly, the body mass index values used to evaluate the MNA-SF were calculated using self-reported data from the participants; thus, such data may not be reliable. Fourthly, semi-mountainous rural regions are characterized by their regional culture, customs, and geographic physical environment; thus, caution must be exercised when making generalizations from these results. Lastly, although an association between malnutrition and oral function in older people has been clarified in earlier reports [41,42], it was not examined in this study. In future studies, it is necessary to examine oral function. Finally, by increasing the number of reports, we believe that common problems and challenges specific to each region will become apparent.

## 5. Conclusions

The nutritional status of single older residents in the semi-mountainous rural region was good overall. However, nutritional status was associated with the following social situation factors: participation in local residents’ association gatherings, social interaction with friends, and psychological state such as being at risk of depression/anxiety. Therefore, to enable older residents living alone in rural areas to maintain a healthy lifestyle, we recommend that community health workers share information on the health condition of residents and promote hosted events such as social activities.

## Figures and Tables

**Table 1:** Binary list of participant characteristics, social situation, environment, and psychological status.

Binary Items	Items in Answer
**Age**	
Early older	65–74
Late-stage older	≥75
**Occupation**	
Retired	Retired
Worker	Self-employed, Part-time, Other
**Period of residence**	
≥20	≥20
<20	10–20, 3–10, 1–3, <1
**Grocery buying frequency**	
One time or less per week	Weekly
Two times or more per week	Two to three times weekly・More than four times per week
**Subjective access to grocery shopping**	
Easy	Easy・Somewhat easy
Difficult	Somewhat difficult・Difficult
**Subjective health**	
Unhealthy	Completely unhealthy・Somewhat unhealthy
Healthy	Somewhat healthy・Healthy
**Subjective happiness**	
Unhappy	Completely unhappy・Somewhat unhappy
Happy	Somewhat happy・Happy
**Financial situation**	
Dire	Very dire・Somewhat dire
Comfortable	Normal・Somewhat comfortable・Very comfortable

**Table 2:** Characteristics of participants (*n* = 53).

	*N*	%
**Sex**		
Male	7	13.2
Female	46	86.8
**Age**		
Early older	7	13.2
Late-stage older	46	86.8
**Occupation**		
Retired	45	84.9
A self-employed business	5	9.4
Part-time	2	3.8
Others	1	1.9
**Period of residence**		
≥20	48	90.6
10–20	1	1.9
3–10	3	5.7
1–3	0	0
<1	1	1.9
**Marital status**		
Married	48	90.6
Never married	5	9.4
**Parental status**		
Children	46	86.8
No child	7	13.2
**Presence or absence of children living within the prefecture**		
Within the same prefecture	36	78.3
Out of prefecture	9	19.6
**Medical history**		
None	8	15.1
Has had:	45	84.9
Hypertension	33	73.3
Diabetes mellitus	6	13.3
Heart disease	5	11.1
Ophthalmic disease	7	15.6
Pulmonary disease	3	6.7
Musculoskeletal diseases	6	13.3
Others	8	17.8

**Table 3:** Relationship between social situation, psychological state, and MNA-SF categories (*n* = 53).

		Normal Nutrition Group (*n* = 38)		Risk of Malnutrition Group (*n* = 15)		*p*-Value
		*n*	%	*n*	%	
**Attributes**						
Sex	Male	5	13.2	2	13.3	1.000
	Female	33	86.8	13	86.7	
Age	Early older	6	15.8	1	6.7	0.658
	Late-stage older	32	84.2	14	93.3	
Occupation	Retired	32	84.2	13	86.7	1.000
	Worker	6	15.8	2	13.3	
Period of residence	≥20	35	92.1	13	86.7	0.614
	<20	3	7.9	2	13.3	
Marital status	Married	35	92.1	13	86.7	0.614
	Never married	3	7.9	2	13.3	
Parental status	Children	33	86.8	13	86.7	1.000
	No child	5	13.2	2	13.3	
Presence or absence of children living within the prefecture	Within the same prefecture	26	81.3	10	76.9	0.704
	Out of prefecture	6	18.8	3	23.1	
Medical history	None	4	10.5	4	26.7	0.202
	Has had	34	89.5	11	73.3	
IADL	Normal	29	76.3	11	78.6	1.000
	Low	9	23.7	3	21.4	
**Social situation**						
Participation						
Seniors’ club activities	No	20	52.6	8	53.3	0.963
	Yes	18	47.4	7	46.7	
Hosted events	No	19	50.0	10	66.7	0.272
	Yes	19	50.0	5	33.3	
Local residents’ association gatherings	No	32	84.2	7	46.7	0.013
	Yes	6	15.8	8	53.3	
Exercise classes	No	34	89.5	12	80.0	0.389
	Yes	4	10.5	3	20.0	
Nutrition classes	No	37	97.4	14	93.3	0.490
	Yes	1	2.6	1	6.7	
Social interaction						
Neighbors	No	17	44.7	7	46.7	0.899
	Yes	21	55.3	8	53.3	
Relatives	No	18	47.4	9	60.0	0.407
	Yes	20	52.6	6	40.0	
Friends	No	19	50.0	13	86.7	0.014
	Yes	19	50.0	2	13.3	
Community health workers	No	28	73.7	13	86.7	0.472
	Yes	10	26.3	2	13.3	
Meal sharing	Yes	11	28.9	6	42.9	0.506
	No	27	71.1	8	57.1	
**Environment**						
Received nutritional information						
Television	No	9	23.7	7	46.7	0.182
	Yes	29	76.3	8	53.3	
Radio	No	36	94.7	15	100.0	1.000
	Yes	2	5.3	0	0.0	
Newspapers/brochures	No	27	71.1	10	66.7	0.751
	Yes	11	28.9	5	33.3	
Neighbors	No	25	65.8	11	73.3	0.748
	Yes	13	34.2	4	26.7	
Word of mouth	No	33	86.8	15	100.0	0.305
	Yes	5	13.2	0	0.0	
Grocery buying frequency	One time or less per week	21	55.3	8	53.3	0.899
	Two times or more per week	17	44.7	7	46.7	
Where grocery buying						
Traveling sales vendors	No	36	94.7	14	93.3	1.000
	Yes	2	5.3	1	6.7	
Supermarkets	No	8	21.1	6	40.0	0.182
	Yes	30	78.9	9	60.0	
Convenience stores	No	37	97.4	15	100.0	1.000
	Yes	1	2.6	0	0.0	
Delivery services	No	34	89.5	11	73.3	0.202
	Yes	4	10.5	4	26.7	
Grocery stores	No	27	71.1	10	66.7	0.751
	Yes	11	28.9	5	33.3	
Means of transport used to go shopping						
On foot	No	33	86.8	15	100.0	0.305
	Yes	5	13.2	0	0.0	
By bicycle	No	36	94.7	12	80.0	0.131
	Yes	2	5.3	3	20.0	
By car	No	28	73.7	9	60.0	0.342
	Yes	10	26.3	6	40.0	
By taxi	No	35	92.1	15	100.0	0.550
	Yes	3	7.9	0	0.0	
By community bus	No	30	78.9	13	86.7	0.706
	Yes	8	21.1	2	13.3	
None	No	35	92.1	13	86.7	0.614
	Yes	3	7.9	2	13.3	
Subjective access to grocery shopping	Easy	28	73.7	7	53.8	0.298
	Difficult	10	26.3	6	46.2	
**Psychological state**						
Subjective health	Unhealthy	8	21.1	7	46.7	0.091
	Healthy	30	78.9	8	53.3	
Subjective happiness	Unhappy	10	28.6	4	28.6	1.000
	Happy	25	71.4	10	71.4	
Financial situation	Dire	3	8.1	2	13.3	0.619
	Comfortable	34	91.9	13	86.7	
Risk of depression/anxiety	No	24	66.7	4	28.6	0.015
	Yes	12	33.3	10	71.4	

*p*-value: chi-square test. Excluding non-answer.

**Table 4:** Results of binomial logistic regression analysis (*n* = 50).

Variables	B	SE	Wald	df	OR	95%CI		*p*-Value
**Social interaction with friends**								
Yes	−2.191	0.975	5.054	1	0.112	0.017	0.755	0.025
**Participation in local residents’ association gatherings**								
Yes	2.004	0.942	4.524	1	7.417	1.170	47.007	0.033
**Risk of depression/anxiety**								
Yes	2.547	0.948	7.211	1	12.769	1.990	81.944	0.007

Dependent variable—normal nutrition group: 0; risk of malnutrition group: 1.

## Data Availability

The datasets used and/or analyzed during the current study are available upon reasonable request to the corresponding author.
